# Coping with rheumatic stressors (CORS) questionnaire: Spanish translation and cross-cultural adaptation

**DOI:** 10.1186/s41687-023-00557-z

**Published:** 2023-02-13

**Authors:** Diego Benavent, Andrea Jochems, Dora Pascual-Salcedo, Gijs Jochems, Chamaida Plasencia-Rodríguez, Sofia Ramiro, Wim van Lankveld, Alejandro Balsa, Victoria Navarro-Compán

**Affiliations:** 1 Department of Rheumatology, Hospital Universitario La Paz, IdiPAZ, Madrid, Spain; 2 Immuno-Rheumatology Group, Hospital Universitario La Paz, IdiPAZ, Madrid, Spain; 3 Promega Biotech Ibérica, Madrid, Spain; 4 Department of Rheumatology, Leiden University Medical Center, Leiden, The Netherlands; 5 Department of Rheumatology, Zuyderland Medical Center, Heerlen, The Netherlands; 6 Musculoskeletal Rehabilitation Research Group, Institute of Health Studies, HAN University of Applied Sciences, 6503 GL Nijmegen, The Netherlands

**Keywords:** axSpA, Coping, Translation, Validation, Questionnaire

## Abstract

**Background:**

Rheumatic and Musculoskeletal Diseases (RMDs) substantially impact the lives of patients, with complex associations between disease severity and self-perceived health status. In this regard, the Coping with Rheumatic Stressors (CORS) questionnaire was developed to measure how patients with RMDs cope with stressors such as pain, limitations or dependency. The CORS is not currently available in Spanish, and therefore the adaptation of this instrument is needed.

**Objective:**

First, to cross-culturally adapt the CORS into Spanish for Spain. Secondly, to test the conceptual equivalence of the translated version in patients with axial spondyloarthritis (axSpA).

**Methods:**

A translation of the CORS into Spanish was performed adhering to the forward-backward procedure described by Beaton. Two translators produced independent forward translations of the item content, response options, and instructions of the CORS into Spanish. Both versions were harmonized in a consensual version. Another translator back-translated the synthesized version into Dutch. A scientific committee including all the translators, one methodologist and a rheumatologist, held a meeting and reached consensus on discrepancies to develop a final draft version of the Spanish CORS. Then, a field test with cognitive debriefing was conducted, involving a sample of 10 patients with axSpA.

**Results:**

The translation process of the CORS was completed after the discussion of some discrepancies throughout the process. The first translation was done without major complications. Back-translation presented some discrepancies. These led to minor modifications in the wording in one response option and 15 questionnaire items. The scientific committee agreed upon a final version of the questionnaire. Cognitive debriefing, led to minor modifications; for example, three respondents indicated that one of the statements in the instructions was syntactically complex (“indique cuán a menudo usted ha llevado a cabo dicho comportamiento”) which led to its adjustment. The process indicated that the final CORS Spanish questionnaire was clear and understandable to all patients.

**Conclusions:**

The Spanish version of the CORS showed good cross-cultural validity and good face validity according to the field test. Before the Spanish CORS is implemented, further validation is in progress to test the psychometric properties of the instrument in patients with axSpA.

**Supplementary Information:**

The online version contains supplementary material available at 10.1186/s41687-023-00557-z.

## Background

Rheumatic and musculoskeletal diseases (RMDs) are a heterogeneous group of conditions that affect bones, joints, muscles, tendons and ligaments which affect hundreds of millions of people worldwide [1] These diseases impact the overall health of the individual, including psychological status [2]. Being complex chronic diseases, RMDs can be emotionally and intellectually challenging. Hence, illness perception and coping might play a relevant role in their evolution and management [3, 4].

“Coping” refers to the cognitive and behavioral strategies an individual employs to manage the specific difficulties associated with daily life and their disease [5]. Coping strategies have been classified according to the dimensions of behavioral coping -which involves the actions a person takes when under pressure- and cognitive coping- which includes emotions and perceptions that people attribute to stressful situations [6]. The most frequent coping strategies in both dimensions can be classified as active and passive, which represent opposed ends of the spectrum. Active coping is focus-oriented and has been linked to adaptative effects, while passive coping is avoidant and has been related to unfavorable effects, including worse functional outcomes [7].

Among the RMDs, axial spondyloarthritis (axSpA) is one of the most relevant diseases associated with chronic stressors, as it may heavily affect functioning in young age through the impairment of spinal mobility and limitations in daily activities [8]. This negative impact on functioning entails a significant burden that may increase feelings of helplessness and negative emotions, which can lead to loss of ability to cope with the difficulties associated with the disease [9, 10]. Concerning coping in patients with axSpA, Boonen et al*. *[11] identified avoidant behavioral strategies to be associated with withdrawal from the work force in patients with ankylosing spondylitis, nowadays also known as radiographic axSpA (r-axSpA). Another study following 90 patients with r-axSpA over a 4-year period showed that avoidant coping at a particular time is independent of disease duration or status[12]. Nonetheless, studies investigating coping strategies of patients with axSpA are scarce.

The Coping with Rheumatic Stressors (CORS) questionnaire assesses coping methods for the three main chronic stressors of rheumatic diseases—pain, limitations, and dependency. It was initially developed and validated in Dutch specifically in rheumatoid arthritis, before being validated in axSpA [12, 13]. While various instruments have been developed to evaluate an individual's coping mechanisms for dealing with pain in recent decades, it is important to emphasize the unique potential of the CORS in the Spanish setting. Some other instruments, such as the Ways of Coping Checklist (WCC), are general in nature [14]. While others such as the Coping Strategies Questionnaire (CSQ) are specific to individuals with chronic pain, they evaluate coping strategies solely related to pain [15]. Unlike this, the CORS evaluates coping strategies also in relation to limitations or dependency, which is necessary to evaluate to obtain information that is relevant for the treatment of patients with axSpA. This includes not only the use of medication or physiotherapy, but also the provision of psychological support that can help patients accept certain limitations and dependency. Besides, there is a lack of questionnaires that specifically evaluate coping strategies in Spanish individuals with chronic pain [16]. These issues led to the cross-cultural adaptation of an instrument that attempts to address these shortcomings.

The purpose of the current study is first, to translate and cross-culturally adapt the CORS into Spanish for Spain and secondly, to assess the translated version in patients with axSpA.

## Methods

**Fig. 1:**
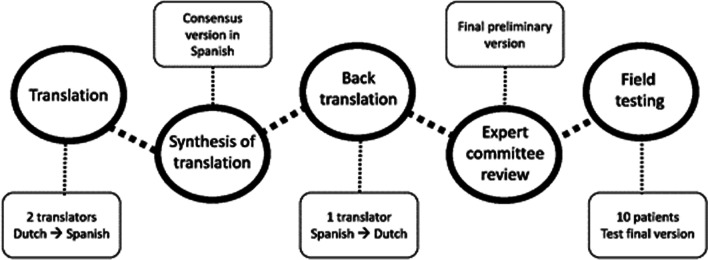
General Coping with rheumatic stressors (CORS) translation and cross-cultural adaptation workflow and procedure

Translation and cross-cultural adaptation of the CORS into Spanish for Spain followed current international recommendations according to the Beaton method [17]. This was performed using the forward-backward procedure, which consists of five sequential steps, as shown in Fig. 1. The project's scientific committee consisted of a methodologist (VN-C), who guided the process of cross-cultural adaptation and ensured methodological consistency, a rheumatologist (DB) and three bilingual Dutch-Spanish translators (AJ, DPS, GJ). The translation and cross-cultural adaptation process took place from April 2021 to December 2021.

### CORS questionnaire

The CORS is an arthritis specific 61-item self-reported questionnaire which assesses eight coping strategies directed at the most relevant stressors in inflammatory rheumatic diseases [13]. It contains three domains which are pain, limitations, and dependency. The three coping with pain subscales (25 items) involve comforting cognitions, decreasing activity, and diverting attention. The three coping with limitation subscales (23 items) involve optimism, pacing, and creative solution seeking. The two coping with dependence subscales (13 items) involve making an effort to accept dependence and showing consideration [18]. Respondents rate the frequency of their use of each particular coping effort on a 4-point Likert-type scale, from "seldom or never" to "very often". The total score is calculated by taking a sum of the items, ranging 61–244, higher scores indicate better coping.

### Translation

The version used for translation was the original Dutch CORS [13]. Two bilingual native Spanish-speakers (AJ, DPS) independently produced a forward translation into Spanish. This included the translation of item content, responses, and instructions. One of the translators had a background in rheumatic diseases and was informed of the questionnaire while the other translator, who did not have a background in rheumatology, was not informed of the conceptual content nor given clinical background on the questionnaire (informed vs. uninformed translator). Each translator prepared an independent written report of the translations indicating any challenges on the procedure.

### Synthesis of translation

The scientific committee, including the two translators, convened an online consensus meeting. The informed and uninformed translations were compared, and a consensus version was created by harmonizing the two translations, after assessing and discussing the differences between the two versions. A written report outlined how the two translations were combined, documenting the issues resolved and specifying the conclusions reached.

### Back translation

This initial Spanish translation of the CORS was back-translated into the original Dutch by another bilingual translator, native Dutch- speaker (GJ), who was unaware of the original form, contents or purpose of the questionnaire. A translation report was created along with the back translation into Dutch.

### Scientific committee review

The scientific committee evaluated the translation and back-translation reports for conceptual, experiential, idiomatic, and semantic comparability. This ensured that everything remained consistent in meaning while maintaining the essence of the Spanish culture. Then, a draft Spanish version of the CORS was created after the agreement of the differences of previous translations.

### Patient interviews and cognitive debriefing

The draft version of the CORS was evaluated in a sample of 10 native Spanish-speaker patients with axSpA. Patients from various age categories, gender and socioeconomic background, were included sequentially in a representative sample from the rheumatology outpatient clinic at the La Paz University Hospital in Madrid. Inclusion criteria involved a diagnosis of axSpA according to the rheumatologist and ability to communicate in Spanish. Patients were excluded if they had conditions that would have affected the evaluation of the questionnaire, such as dementia or illiteracy.

Patients completed the CORS questionnaire, and were then invited to a one-to-one remote or on-site meeting for cognitive debriefing with a rheumatologist (DB). The purpose of the cognitive debriefing interviews was to assess how well the patients comprehended the questionnaire. In addition, they were inquired about the acceptability of the items to detect potential confusing expressions or items. Each respondent completed the CORS and was questioned about the items and responses. In order to ensure that the translated version was equivalent to the original questionnaire in a practical situation, the meaning of conflicting items was thoroughly evaluated. The goal of the exercise, as indicated to the respondents, was to test the questionnaire rather than have them evaluate themselves.

## Results

The CORS was translated and cross-culturally adapted into Spanish for Spain (Additional file 1).

### Translation of the Dutch CORS original version

The first translation was done without major complications, as both translators reported similar independent forward translations for most items. Hence, consensus on the first translation was reached in the initial meeting. Only minor discrepancies arose between these first independent translations, mainly related to the fact that one of the translators used more formal wording while the other adapted some details to facilitate interpretation. However, both translators agreed that most of the translated statements represented the same aspects. An example of minor discrepancy is the term “omgaan” (to handle), which generated discussion about the actual Spanish translation. One of the translators proposed “afrontar”, whereas the other proposed “lidiar”. After the assessment of the different options, it was agreed that the word that probably best reflects the conceptual meaning and is more used in Spanish would be “convivir”. Likewise, the title arose some doubts in the translators, since both the terms “coping” and “rheumatic stressors” do not present a clear direct translation in Spanish. Likewise, it was agreed that also the word “convivir” reflects the term “coping” while “estresores reumáticos” reflects “rheumatic stressors”; hence the title that was determined was “Convivir con estresores reumáticos”. Nevertheless, it was decided that the acronym of the title of the English version (i.e. CORS) would prevail. As another conflicting example, the structure “Ik probeer” (“I am trying to”) was translated both as “Intento que” or “Trato que”. A consensus was reached to maintain “Intento que” for the whole questionnaire, since it seemed more suitable in this context. Similar to that, all differences were resolved, and a preliminary draft Spanish translation was produced.

### Back translation of the preliminary version of the Spanish CORS

A few discrepancies appeared in the back-translation. These led to minor modifications in the wording in one response option (“muchas veces” to “muy a menudo”) and 15 questionnaire items. As an example of these, “Ik ga de deur uit”, literally meaning “I go out by the door”, was initially translated as such (“salgo por la puerta”); however, it conceptually represents “I go away”, and it was adapted as such (“me voy a la calle”). Some statements required several iterations before reaching consensus; “Ik houd me voor dat sommige mensen er erger aan toe zijn” (“I keep in mind that some people are worse off”), was initially presented as “Me imagino que otras personas están peor que yo”; in the first iteration it was adapted “Me digo a mi mismo que otras personas lo tienen peor”. However, after reviewing these items in context, it was decided that “Pienso que otras personas están peor que yo” better reflected the equivalence of the term with a more adequate formulation. “Ik neem rust door te gaan zitten of liggen” (“I rest by sitting or lying down”), was initially translated as “Descanso yendo a sentarme o tumbarme”-which is the literal translation; however, all the members of the committee agreed that “Descanso sentándome o tumbándome” adapted better. After the discussion and adjustment of the different items, it was agreed that the back-translation presented semantic, experiential and conceptual equivalence with the original version.

### Cognitive debriefing

Ten patients with axSpA tested the final Spanish version of the CORS and were interviewed for cognitive debriefing. Concerning the characteristics of these participants, mean age (SD) was 38.9 (14.4) years; 7 patients were male, while 3 were female. Seven patients had university studies and 6 were actively working. Mean axSpA duration since diagnosis was 11 years; six patients had radiographic axSpA and four non-radiographic axSpA; 6 patients were on biological disease modifying antirheumatic drugs.

Most patients found the questionnaire clear and confirmed the equivalence of all the items. Cognitive debriefing queries and final decisions from the expert committee can be seen in Table 1. Three respondents indicated that one of the statements in the instructions was syntactically complex (“indique cuán a menudo usted ha llevado a cabo dicho comportamiento”) which led to its adjustment (“indique la frecuencia con la cual usted ha tenido dicho comportamiento”). Likewise, two respondents commented that the meaning of the item “Ik houd rekening met anderen” (“I am considerate of others”) was not clear; it was initially translated as “Tengo en cuenta a los demás”, and subsequently adapted in the final version as “Tengo en consideración a los que me ayudan/cuidan”. Some other minor queries did not lead to any change, and the previous formulation remained as initially agreed by the scientific committee. As an example, “Me voy a tiempo a descansar” (translated from “Ik rust op tijd uit”), was discussed among the scientific committee following the comment of a respondent on the potential ambiguity of the statement. However, after assessing different options (such as such as “Me tomo un descanso a tiempo”, “Hago un descanso a tiempo”) this was considered the one that had a best equivalence with the original questionnaire while maintaining an adequate clear and concise language structure. In conclusion, the participants regarded the items of the CORS in Spanish language to be generally clear and intelligible, providing evidence of content validity of the questionnaire.

**Table 1:** Cognitive debriefing queries and final decisions from the expert committee

Original Dutch item	Spanish translation pre-final	# Patient queries	Queries	Final version
*(….) aan te geven hoe vaak u het beschreven gedrag uitvoert*	*(…) indique cuán a menudo usted ha llevado a cabo dicho comportamiento*	3	Literal discrepancies	*(…) indique la frecuencia con la cual usted ha tenido dicho comportamiento*
*Ik rust op tijd uit*	*Me voy a tiempo a descansar*	1	Literal discrepancies	No changes implemented
*Ik probeer er het beste van te maken*	*Intento aprovechar al máximo*	1	Literal discrepancies	No changes implemented
*Ik houd rekening met anderen*	*Tengo en cuenta a los demás*	2	Meaning doubts	*Tengo en consideración a los que me ayudan/cuidan*

## Discussion

The CORS questionnaire was translated and culturally adapted into Spanish for Spain in accordance with the current international guidelines for this procedure [15, 16]. The language equivalence and cultural adaptation were ensured through the translation and back-translations process. Thus, the initial translation was only slightly modified after the back-translation and patients’ interviews. The cognitive debriefing indicated strong content validity as no patients found the items to be irrelevant or confusing.

The development of coping mechanisms is crucial for an adequate management of RMDs. Coping mechanisms may be influenced by a patient's beliefs and understanding of their condition. It is therefore relevant to assess these mechanisms for a holistic management of the disease. While coping may seem like a personal characteristic, it is often shaped by external factors. Thus, most people develop customized coping mechanisms in response to specific circumstances, which can also influence their perception of the disease [9]. Particularly in patients with axSpA, certain coping strategies such as decreasing physical activity because of back pain have shown to present a negative influence on quality of life [19]. In contrast to previous hypothesis considering coping strategies may change between an early and late phase of a disease, it has been shown that coping strategies in patients with axSpA remained remarkably stable over a 2-year follow up [20, 21]. Besides, the way patients cope with pain and limitations has been related to their well-being, and it is therefore of clinical relevance; the importance of coping strategies in the management of RMDs cannot be overlooked [13]. Despite the potential benefit that interventions aiming at improvement of coping strategies, studies evaluating coping in RMDs are scarce, and a lack of coping measurement instrument may be one of the reasons.

Assessment of coping may be performed through patient-reported outcomes (PROs), which evaluate disease outcomes from the patient perspective [22]. In patients with axSpA, PROs are regularly used to assess health status, functional ability or disease activity. However, it has not been clearly established which is the most adequate instrument to assess coping in these in patients [23]. A systematic review by Banerjee et al. [24], which appraised outcome measures for self-management in patients with chronic pain, identified 3 instruments to evaluate coping with pain. The Coping Strategies Questionnaire is a self-reported questionnaire designed to assess six cognitive coping strategies (ignoring pain, reinterpretation, diverting attention, self-statements, catastrophising, praying/hoping) and two behavioral coping responses to pain (increasing activity and increasing pain behavior), that has been used in rheumatoid arthritis [15, 25]. The Chronic Pain Coping Inventory was validated in chronic pain population to measure also cognitive and behavioral coping [26]. The Pain Coping Inventory measures three active coping strategies (transformation, distraction and reducing demands) and three passive coping strategies (ruminating, retreating and resting) [27]. Unlike these scales, the CORS has the advantage that it measures coping styles directly related to disease-specific stressors; besides pain, it also assesses the effect of other stressors such as limitation or dependence [28]. Thus, the assessed styles of coping in the CORS are related to the spectrum of cognitive and behavior responses that may appear following the development of a RMD.

The lack of language availability of some instruments may hinder the assessment of PROs. For this matter, cross-cultural validation ensures an adequate reflection of the translation of an instrument as compared with the original version [29]. Cross-cultural validation requires a standardized process to provide conceptual, experiential, idiomatic and semantic equivalence to the translation of the instrument. Although there are different guidelines and methodologies for cross-cultural validation, Beaton method, which includes “forward-backward” translation and field testing, has proven to be a valid and reliable methodology [17, 30]. We followed this five-step process of translation and back-translation, including a field testing of the final draft translated version of the CORS. This ensured the adequate adaptation of the original version to Spanish culture and idiomatic expressions. It is noteworthy that Spanish is the second most widely spoken mother tongue in the world after Chinese, and it is used as well in many nations as a second language.

Nevertheless, our study has some limitations. Ten participants were involved in the field test, and a higher number may have provided more insights in cognitive debriefing. However, the sample was representative in terms of gender, age and educational level of the population with axSpA. Although an optimal number for cognitive debriefing has not been defined, the field test of the health index for axSpA recently developed by Assessment of SpondyloArthritis International Society (ASAS) included a similar number of patients for a translation in 15 languages [31]. Of note, this instrument has shown very good psychometric properties in these translations. Besides, only patients with a particular disease in the whole the spectrum of RMDs have been included, and therefore cross-cultural validation for other diseases was not assessed. Only having one back-translator might be considered limited, but the whole process included three translators that allowed a robust discussion and consensus. Another limitation was that participants involved in the translation were all from Spain, which ensures content validity in Spanish from Spain, but may not account for linguistic variations in some items in other regions of the world. Further research in some other Spanish-speaking countries is needed to confirm the validity of this Spanish version of the CORS. Besides, it is relevant to highlight that this study serves as the first stage in the instrument's full validation. Hence, before the Spanish CORS can be used in clinical practice, it is essential to evaluate its psychometric qualities in a wider patient population.

## Conclusion

The CORS questionnaire was successfully translated into Spanish, demonstrating sufficient face and content validity for the assessment of coping in patients with axSpA. However, before putting this instrument to use in clinical practice, further steps toward full validation of its psychometric properties are required.

## Supplementary Information

**Additional file 1**. Questionnaire.

## Data Availability

The data underlying this article will be shared on request to the corresponding author with permission of the scientific Committee of the project.

## Abbreviations

ASAS: Assessment of SpondyloArthritis International Society
axSpA: Axial spondyloarthritis
CORS: Coping with Rheumatic Stressors
PROs: Patient-reported outcomes
r-axSpA: Radiographic axSpA
RMDs: Rheumatic and musculoskeletal diseases
