# Supplementary material for: Coping with rheumatic stressors (CORS) questionnaire: Spanish translation and cross-cultural adaptation
Source: J Patient Rep Outcomes. 2023 Feb 13;7:11. doi: 10.1186/s41687-023-00557-z (PMC9925618; doi:10.1186/s41687-023-00557-z)
Supplement: Supplementary file 1 — Additional file 1. Questionnaire. [file 41687_2023_557_MOESM1_ESM.docx]

**Cuestionario CORS**

1. **Convivir con el dolor:**

Las personas que tienen dolor desarrollan diferentes maneras de convivir con él. A continuación se muestran una serie de afirmaciones acerca de lo que usted puede llegar a hacer o pensar cuando experimenta dolor.

Le pedimos que después de cada afirmación, indique la frecuencia con la cual usted ha tenido dicho comportamiento. Marque la casilla adecuada.

No se piense mucho la respuesta, pero asegúrese de responder a todas las preguntas.

| **¿Qué hace usted cuando experimenta dolor?** | **Ocasionalmente/**  **Nunca** | **A veces** | **A menudo** | **Muy a menudo** |
| --- | --- | --- | --- | --- |
| 1. Creo que el dolor es soportable |  |  |  |  |
| 1. Dejo de hacer mis ocupaciones |  |  |  |  |
| 1. Me concentro en otra cosa |  |  |  |  |
| 1. Pienso que el dolor irá a menos |  |  |  |  |
| 1. Me limito a ocupaciones sencillas |  |  |  |  |
| 1. Pienso en cosas o acontecimientos agradables |  |  |  |  |
| 1. Me imagino que a partir de ahora irá mejor |  |  |  |  |
| 1. Interrumpo mis ocupaciones con una pausa para descansar |  |  |  |  |
| 1. Busco compañía |  |  |  |  |
| 1. Intento no dar importancia al dolor |  |  |  |  |
| 1. Retomo el trabajo cuando me encuentro mejor |  |  |  |  |
| 1. Me voy a dar un paseo |  |  |  |  |
| 1. Me imagino que otras personas están peor |  |  |  |  |
| 1. Busco distraerme con un libro, música, un programa de televisión, o algo similar |  |  |  |  |
| 1. Me pongo a hacer algo que me gusta |  |  |  |  |
| 1. Intento mantenerme optimista |  |  |  |  |
| 1. Me aseguro de no tener que hacer ningún esfuerzo físico |  |  |  |  |
| 1. Me ocupo en algo para olvidar el dolor |  |  |  |  |
| 1. Intento no pensar en el dolor |  |  |  |  |
| 1. Descanso sentándome o tumbándome |  |  |  |  |
| 1. Me entretengo con mis aficiones |  |  |  |  |
| 1. Intento disfrutar a pesar del dolor |  |  |  |  |
| 1. Me voy a la cama |  |  |  |  |
| 1. Me pongo a hacer algo para no sentir el dolor |  |  |  |  |
| 1. Intento mantener el ánimo |  |  |  |  |

1. **Convivir con las limitaciones:**

Las personas con limitaciones físicas tienen diferentes maneras de convivir con las mismas. A continuación, se muestran diferentes maneras mediante las cuales las personas conviven con sus limitaciones.

Le pedimos que después de cada afirmación, indique la frecuencia con la cual usted ha tenido dicho comportamiento. Marque la casilla adecuada.

No se piense mucho la respuesta, pero asegúrese de responder a todas las preguntas.

|  | **Ocasionalmente/**  **Nunca** | **A veces** | **A menudo** | **Muy a menudo** |
| --- | --- | --- | --- | --- |
| 1. Me contento con lo que sí puedo hacer |  |  |  |  |
| 1. Evito el trabajo pesado |  |  |  |  |
| 1. Busco nuevas ocupaciones |  |  |  |  |
| 1. Intento ver el lado positivo de la situación |  |  |  |  |
| 1. Tengo en cuenta mis limitaciones |  |  |  |  |
| 1. Busco soluciones para mis limitaciones |  |  |  |  |
| 1. Pienso que otras personas están peor que yo |  |  |  |  |
| 1. Lo que tenga que hacer, lo hago adaptado a mis limitaciones |  |  |  |  |
| 1. Intento hacer de todo |  |  |  |  |
| 1. Intento mantenerme optimista |  |  |  |  |
| 1. Me tomo mi tiempo para mis ocupaciones |  |  |  |  |
| 1. Pienso en maneras que me permitan hacer mis cosas |  |  |  |  |
| 1. Intento confiar en el futuro |  |  |  |  |
| 1. Me convenzo de que no tengo que acabar todas las tareas obligatoriamente |  |  |  |  |
| 1. Valoro de qué manera puedo organizar mejor mis ocupaciones |  |  |  |  |
| 1. Distribuyo mis ocupaciones a lo largo del día |  |  |  |  |
| 1. Busco la manera en la que, a pesar de todo, pueda hacer algo |  |  |  |  |
| 1. Hago mis tareas con descansos |  |  |  |  |
| 1. Invento nuevas maneras para poder hacer mis tareas |  |  |  |  |
| 1. Me voy a tiempo a descansar |  |  |  |  |
| 1. Si algo no me sale, lo hago de otra manera |  |  |  |  |
| 1. Dejo mis actividades a tiempo |  |  |  |  |
| 1. Dejo el trabajo pesado para otros |  |  |  |  |

1. **Convivir con la dependencia:**

A continuación, se muestran diferentes maneras empleadas por las personas para manejar su dependencia de otros.

Le pedimos que después de cada afirmación, indique la frecuencia con la cual usted ha tenido dicho comportamiento. Marque la casilla adecuada.

No se piense mucho la respuesta, pero asegúrese de responder a todas las preguntas.

|  | **Ocasionalmente/**  **Nunca** | **A veces** | **A menudo** | **Muy a menudo** |
| --- | --- | --- | --- | --- |
| 1. Me convenzo de que ahora las cosas son así. |  |  |  |  |
| 1. Trato de ser útil para otros |  |  |  |  |
| 1. Intento admitir mi dependencia |  |  |  |  |
| 1. Intento aprovechar al máximo |  |  |  |  |
| 1. Acepto mi dependencia |  |  |  |  |
| 1. Tengo en consideración a los que me ayudan/cuidan |  |  |  |  |
| 1. Intento estar en paz con mi dependencia |  |  |  |  |
| 1. Me adapto a los demás |  |  |  |  |
| 1. Intento no agobiarme por mi dependencia |  |  |  |  |
| 1. Trato de no ser una carga para los demás |  |  |  |  |
| 1. Asumo mi dependencia |  |  |  |  |
| 1. Intento hacer algo a cambio |  |  |  |  |
| 1. Intento no pedir demasiado a una misma persona |  |  |  |  |
